# Temporal Triangular Alopecia—Clinical and Dermoscopic Features of a Rare Entity

**DOI:** 10.3390/diagnostics15202621

**Published:** 2025-10-17

**Authors:** Beata Zagórska, Jakub Żółkiewicz, Urszula Maińska, Martyna Sławińska

**Affiliations:** 1 Department and Clinic of Dermatology, Venerology and Allergology, Medical University of Gdansk, 80-214 Gdansk, Poland; 2 Department and Clinic of Dermatology, Venerology and Allergology, University Clinical Center, 80-214 Gdansk, Poland

**Keywords:** TTA, temporal triangular alopecia, dermoscopy

## Abstract

We present the case of an 18-year-old female patient who attended the Dermatology Outpatient Clinic for a routine total body examination. Clinical evaluation revealed a triangular, non-scarring alopecic patch in the left temporal region, which the patient reported had been present since birth. Based on clinical and trichoscopic findings, a diagnosis of temporal triangular alopecia (TTA) was established. Given the non-progressive nature of the condition and the absence of any impact on quality of life, no treatment was initiated at the patient’s request.

**Figure 1:**
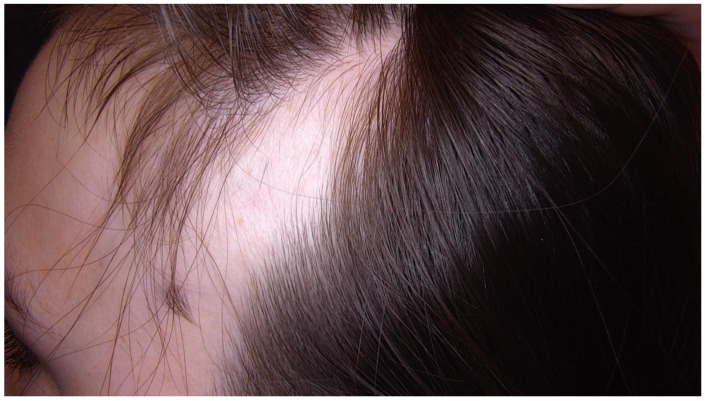
An 18-year-old female patient presented to the Dermatology Outpatient Clinic for a routine total body examination. During dermoscopic evaluation of the scalp, a triangular patch of alopecia was observed in the left temporal region. According to the patient, this lesion had been present since birth. Her medical history was notable for multiple congenital anomalies, including scoliosis, rib fusion, iron deficiency anemia, and a secundum atrial septal defect (ASD II). Her regular medications included esomeprazole, vitamin D3, and biotin. She had also undergone surgical correction for congenital esophageal atresia. In view of the constellation of congenital defects, the patient was referred to a genetician and is currently awaiting specialist evaluation.

**Figure 2:**
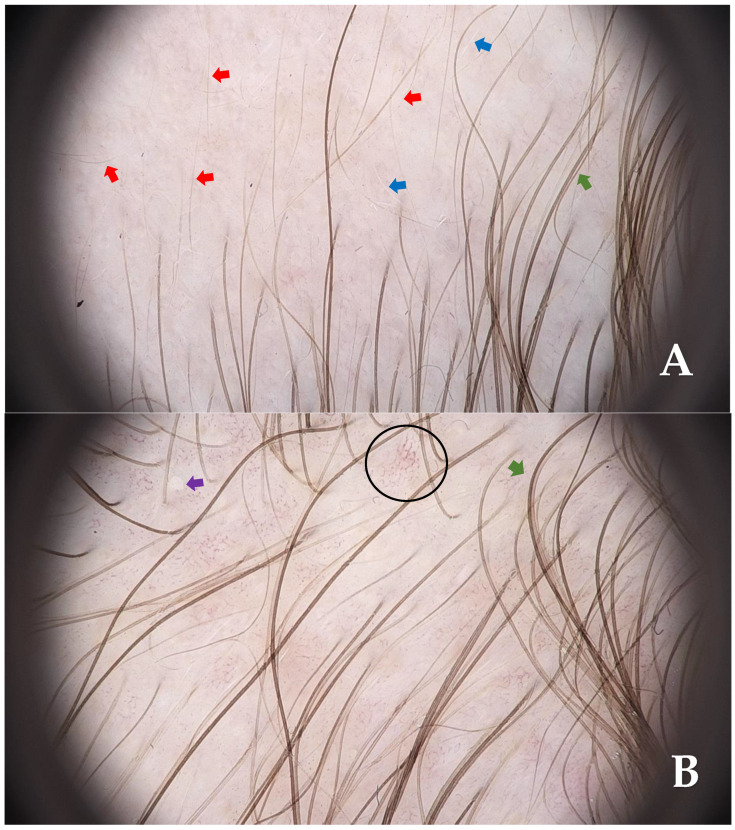
Videodermoscopy revealed the presence of vellus hairs (red arrows), white vellus hairs (blue arrows) (**A**), and isolated empty follicular openings (purple arrow) surrounded by terminal hairs (green arrow) in the clinically unaffected area of the temporal scalp. Additionally, polymorphic dilated blood vessels were observed (black circle) ((**B**), FotoFinder Vexia Medicam 800 HD; 20× magnification; non-polarized light; immersion: ultrasound gel). Based on clinical examination and dermoscopic findings, a diagnosis of temporal triangular alopecia (TTA) was established. TTA, also known as congenital triangular alopecia or Brauer’s nevus, is a rare and benign condition. It is characterized by a well-demarcated, localized patch of non-cicatricial and non-inflammatory hair loss [1]. TTA was first described by Sabouraud in 1905 under the term ‘alopecia triangulaire congénitale de la temp’ [2]. Clinically, it presents as a nonprogressive, well-circumscribed patch of alopecia, most commonly triangular, oval, or lanceolate in configuration. The condition is most often unilateral, with a notable predilection for the left frontotemporal region [3]. While typically confined to this anatomical site, less common involvement of the temporoparietal, midfrontal, or occipital areas has also been reported [4,5]. Despite the unclear pathogenesis of TTA, a paradominant pattern of inheritance has been proposed, supported by both sporadic and familial case reports. Associations with syndromic conditions such as Down syndrome, LEOPARD syndrome, Dandy–Walker malformation, wooly hair, and other congenital anomalies have been described, suggesting a possible developmental or mosaic genetic component [2,6]. TTA most often presents between 2 and 9 years of age, although occasionally it may be present from birth or manifest in adulthood [7]. According to the available literature, epidemiological data on TTA remain limited. To date, only a single publication has proposed an estimated prevalence of approximately 0.11% in the general population [8]. While diagnosis is primarily clinical, trichoscopic assessment may serve as a valuable adjunct in uncertain cases. Characteristic dermoscopic features include short, hypopigmented vellus hairs, upright regrowing hairs, empty follicles, white dots and, less frequently, arboriform vascular pattern, pigtail or circle hairs within the alopecic area, which is typically surrounded by normal terminal hairs [7,9,10]. In cases where the clinical diagnosis remains uncertain, histopathological evaluation may be considered. Such analysis typically reveals a preserved follicular density with a predominance of vellus hairs, a relative paucity of terminal follicles, and no histological evidence of inflammation or fibrosis [3]. In the case described herein, histopathological confirmation was not pursued, as the constellation of clinical and dermoscopic features was entirely consistent with the diagnostic characteristics of temporal triangular alopecia as delineated in the existing literature. High-resolution videodermoscopy (HRVD) combined with linear field confocal optical coherence tomography (LC-OCT) is another method that may support non-invasive diagnostics of TTA [11]. Dermoscopic findings, such as the predominance of vellus hairs and preserved follicular openings, correlate closely with the findings revealed in LC-OCT examination. LC-OCT of TTA shows normal epidermal, dermal, and adnexal structures. Only minor follicular changes are observed, with the absence of scarring [11]. TTA is often misdiagnosed as other forms of localized non-scarring alopecia, including androgenic alopecia, alopecia areata, trichotillomania, traction alopecia, tinea capitis or aplasia cutis congenita [3,12]. Misdiagnosis often leads to unnecessary and ineffective treatments. Recognition of characteristic clinical and trichoscopic features is therefore crucial for accurate diagnosis and avoidance of invasive procedures. To date, there is no effective pharmacological treatment, although surgical excision and hair transplantation have been used in selected cases [13,14,15,16]. Platelet-rich plasma has been investigated experimentally, with initial dermoscopic improvement observed over a 5-month period, though no sustained clinical benefit was noted, and hair loss recurred within 6 months following treatment cessation [17]. Additionally, topical application of 5% minoxidil has shown promising results in isolated reports, demonstrating early regrowth of terminal hairs within the alopecic area, as confirmed by trichoscopy, although maintenance of therapeutic effect appears contingent on continued use [18,19,20]. A comprehensive summary of the key clinical, dermoscopic, histopathological, and therapeutic aspects of TTA is provided in Supplementary Table S1.

## Data Availability

All relevant data are within the manuscript.

## Supplementary Materials

The following supporting information can be downloaded at https://www.mdpi.com/article/10.3390/diagnostics15202621/s1, Table S1: Summary of key clinical, dermoscopic, histopathological, and therapeutic aspects of TTA.
