# Supplementary material for: Temporal Triangular Alopecia—Clinical and Dermoscopic Features of a Rare Entity
Source: Diagnostics (Basel). 2025 Oct 17;15(20):2621. doi: 10.3390/diagnostics15202621 (PMC12564162; doi:10.3390/diagnostics15202621)
Supplement: Supplementary file 1 [file diagnostics-15-02621-s001.zip › diagnostics-3878151-supplementary.pdf]

Supplementary Table S1. Summary of key clinical, dermoscopic, histopathological, and therapeutic aspects of TTA

Domain	Key Features
**Synonyms**	Congenital triangular alopecia, Brauer's nevus
**Epidemiology**	Estimated prevalence ~0.11% (limited data)
**Onset**	Most often between 2–9 years; may be present at birth or manifest in adulthood
**Diagnosis**	Primarily clinical, supported by trichoscopy; histopathology or HRVD/LC-OCT used in uncertain cases
**Clinical presentation**	Nonprogressive, well-circumscribed patch of alopecia; typically triangular/oval/lanceolate; usually unilateral (predilection for left frontotemporal region)
**Associations**	Down syndrome, LEOPARD syndrome, Dandy-Walker malformation, woolly hair, and other congenital anomalies
**Dermoscopic features**	Short hypopigmented vellus hairs, upright regrowing hairs, empty follicles, white dots
**Histopathology**	Preserved follicular density; predominance of vellus hairs; reduced terminal follicles; absence of inflammation or fibrosis
**Differential diagnosis**	Androgenic alopecia, alopecia areata, trichotillomania, traction alopecia, tinea capitis, aplasia cutis congenita
**Treatment**	No consistently effective pharmacological treatment; options: surgical excision, hair transplantation; experimental: PRP (transient treatment effect), topical 5% minoxidil (initial regrowth, requires continuous use).
